# Alternative splicing discriminates molecular subtypes and has prognostic impact in diffuse large B-cell lymphoma

**DOI:** 10.1038/bcj.2017.71

**Published:** 2017-08-25

**Authors:** S-K Leivonen, M Taskinen, A Cervera, M-L Karjalainen-Lindsberg, J Delabie, H Holte, R Lehtonen, S Hautaniemi, S Leppä

**Affiliations:** 1 Research Program Unit, Medical Faculty, University of Helsinki, Helsinki, Finland; 2 Department of Oncology, Helsinki University Hospital Comprehensive Cancer Center, Helsinki, Finland; 3 Department of Pathology, Haartman Institute, University of Helsinki and Helsinki University Hospital, Helsinki, Finland; 4 Department of Pathology, Oslo University Hospital, Oslo, Norway; 5 Department of Oncology, Oslo University Hospital, Oslo, Norway

## Abstract

Effect of alternative splicing (AS) on diffuse large B-cell lymphoma (DLBCL) pathogenesis and survival has not been systematically addressed. Here, we compared differentially expressed genes and exons in association with survival after chemoimmunotherapy, and between germinal center B-cell like (GCB) and activated B-cell like (ABC) DLBCLs. Genome-wide exon array-based screen was performed from samples of 38 clinically high-risk patients who were treated in a Nordic phase II study with dose-dense chemoimmunotherapy and central nervous system prophylaxis. The exon expression profile separated the patients according to molecular subgroups and survival better than the gene expression profile. Pathway analyses revealed enrichment of AS genes in inflammation and adhesion-related processes, and in signal transduction, such as phosphatidylinositol signaling system and adenosine triphosphate binding cassette transporters. Altogether, 49% of AS-related exons were protein coding, and domain prediction showed 28% of such exons to include a functional domain, such as transmembrane helix domain or phosphorylation sites. Validation in an independent cohort of 92 DLBCL samples subjected to RNA-sequencing confirmed differential exon usage of selected genes and association of AS with molecular subtypes and survival. The results indicate that AS events are able to discriminate GCB and ABC DLBCLs and have prognostic impact in DLBCL.

## Introduction

Diffuse large B-cell lymphoma (DLBCL) is the most common lymphoid neoplasm in adults, comprising 30–40% of all malignant lymphomas. It is an aggressive disease, where ∼60% of patients can be cured with a combination of rituximab and anthracycline-based CHOP or CHOP-like chemoimmunotherapy.^1, 2^ However, responses to treatment are still largely unpredictable, and survival of the patients, who experience disease relapse or have primary refractory disease, is dismal. Thus, there is a growing need to further understand the molecular mechanisms underlying the disease that would not only provide information to predict the survival, but also enable the design of more targeted therapeutic strategies.

Based on gene expression profiling, three molecular subtypes showing germinal center B-cell (GCB), activated B-cell (ABC) and primary mediastinal B-cell lymphoma signatures have been identified. These subtypes differ in their genotypic, phenotypic and clinical features.^3, 4, 5, 6^ During the past few years, studies applying next-generation sequencing techniques have provided further insights into the heterogeneity and pathogenesis of DLBCL.^7, 8, 9^ Especially, a number of genetic alterations have been shown to be characteristic of GCB or ABC subtypes. However, despite the rapidly growing number of genetic aberrations reported in DLBCL, association of these findings with treatment outcome remains to be shown.

Alternative splicing (AS) is a common regulatory mechanism generating multiple RNA transcripts from a single gene and allowing enormous functional diversity in protein isoforms. The vast majority of the human genes, evaluates ranging from 70 to 95%, are alternatively spliced.^10^ Growing evidence suggests that AS is closely associated with cancer pathogenesis and progression.^11, 12, 13^ Alternatively spliced signatures derived from gene expression profiling have been shown to be more reliable for diagnostic purposes than signatures derived from expression profiling, for example in prostate cancer.^14^ Furthermore, isoform-level expression profiles can discriminate cancer cell lines from noncancer cells better than gene-level expression profiles.^15^

In DLBCL, the role of AS remains largely unexplored. Previous studies have mainly focused on AS isoforms of individual genes, such as *FOXP1*, where contradictory findings concerning its prognostic role can partly be explained by smaller and potentially oncogenic *FOXP1* isoforms primarily expressed in ABC-type DLBCL.^16^ Other previously reported genes with AS in DLBCL include a developmentally regulated B-lymphoid phosphatase, *PTPRO*, regulating G_0_/G_1_ arrest^17^ and a leukocyte homing and hyaluronidase receptor *CD44*.^18, 19, 20, 21^

In this study, we have evaluated genome-wide gene and exon expression profiles in DLBCL. Differential exon usage was found to be a common event in DLBCL. Splicing events affected pivotal genes involved in DLBCL signaling, and were able to discriminate the patients according to cell of origin (COO) and survival.

## Materials and methods

### Patients

Prospectively collected discovery cohort consisted of 38 DLBCL patients <65 years old with clinically high-risk (age-adjusted International Prognostic Index Score 2–3) disease (Table 1). The patients were treated in a Nordic phase II NLG-LBC-04 protocol with six courses of dose-dense R-CHOEP-14 chemoimmunotherapy followed by systemic central nervous system prophylaxis with high-dose methotrexate and high-dose cytarabine.^22^ Further information on the patient cohort is provided in the Supplementary Materials and methods. The protocol and sampling were approved by Institutional Review Boards, National Medical Agencies and Ethics Committees in Finland, Denmark, Sweden and Norway, and the trial was registered at ClinicalTrials.gov, identifier number NCT01502982. All patients gave informed consent.

Validation cohort consisted of RNA sequencing (RNAseq) and clinical data from 92 R-CHOP-like chemotherapy-treated DLBCL patients generated by the National Cancer Institute (NCI) Cancer Genome Characterization Initiative (CGCI; dbGaP database applied study accession: phs000532.v3.p1)^7, 8^ (Table 1).

### Samples and gene/exon expression analysis

Total RNA was extracted with Qiagen (Hilden, Germany) AllPrep DNA/RNA/Protein Mini kit and examined using Affymetrix Human Exon 1.0 ST arrays according to the manufacturer’s instructions (Affymetrix, Santa Clara, CA, USA). Hybridization protocols and raw expression microarray data are available at ArrayExpress archive (http://www.ebi.ac.uk/arrayexpress/experiments/E-MEXP-3488). Gene- and exon-level expression data for the NLG-LBC-04 discovery cohort were quantified by MEAP (Multiple Exon Array Preprocessing) algorithm^23^ with MEAP probe annotation version 70.

In the CGCI validation cohort, adaptors and low-quality bases were trimmed from the samples before alignment. The reads were aligned with TopHat 2.0.8b using the hg19 human reference genome with Ensembl gene annotation version 70. HTSeq was then used to obtain the exon- and gene-level read counts. Differential expression at exon and gene levels was estimated using DEXSeq (version 1.7.14) and DESeq2 (version 1.4.5), respectively (https://bioconductor.org/packages/3.5/bioc/).

### Molecular subgroup prediction

Samples with exon array data were classified into GCB, ABC and nonclassified subgroups using the gene predictor from Lymphochip data as previously described.^24^ More details are provided in the Supplementary Materials and methods.

### Pathway and GO analysis

KEGG (Kyoto Encyclopedia of Genes and Genomes) pathway functional enrichment analyses were performed with the Pathway-Express (Intelligent Systems and Bioinformatics Laboratory, Detroit, MI, USA). Only enrichment results with false discovery rate <0.05 were considered significant. Gene annotation and gene ontology (GO) term enrichment for biological processes was performed with Gene Set Enrichment Analysis (GSEA)^25^ for the 289 genes with differentially expressed exons (DEEs) log2 fold change >1.5. This resulted into altogether 38 separate ontology terms with false discovery rate <0.05. To reduce the number of GO terms, we regrouped the related ontologies into 9 larger groups.

### Statistical analyses

Data were analyzed using IBM SPSS Statistics 22.0 (IBM, Armonk, NY, USA). *P*-values of <0.05 were considered statistically significant and all *P*-values are two tailed. The χ^2^ test was used to assess the differences in the frequency of the prognostic factors. Cox univariate and multivariate analyses were performed to study the prognostic value of the factors. Kaplan–Meier method was used to estimate survival rates and their differences were compared with log-rank test. Overall survival (OS) was determined from the date of study entry or diagnosis until the last follow-up or death from any cause. Disease-specific survival was calculated as a period between the registration date and the date of death due to lymphoma. Progression-free survival (PFS) was measured as the period between the date of registration or diagnosis and progression or death from any cause. OS, disease-specific survival and PFS were reported in months. A web-based cutoff finder tool at http://molpath.charite.de/cutoff analysis^26^ was used to determine the most prognostic cutoff level for survival outcomes.

### Exon-specific domain identification

DEEs were annotated by their genomic locations (5′ untranslated region (UTR), 3′ UTR, coding, noncoding and unknown). Domain analysis was done for all coding DEEs by translating coding exonic regions into peptide sequences using Ensembl API (version 70) and fetching domain information (Pfam, Smart, SignalP and TMHMM) for all peptide sequences with InterProScan (version 5).^27^ Phosphorylation sites within peptide sequence of each exon were searched against all known phosphorylation motifs downloaded from the PhosphoSitePlus.^28^

## Results

### Clinical characteristics of the patients

Baseline characteristics of the discovery cohort of 38 patients treated in the NLG-LBC-04 protocol^22^ are presented in Table 1. Median age of the patients was 55 years (range 21–65 years). During the median follow-up time of 66 months, 9 patients had relapsed and a total of 9 died. Four of the deaths were not lymphoma related. The estimated 5-year OS, disease-specific survival and PFS rates were 76%, 82% and 74%, respectively. In the CGCI validation cohort of 92 patients, the median age was 61 years (range 17–75 years). During the follow-up time of 59 months, 24 patients had relapsed and 21 died. Of the deaths, three were not lymphoma related. The estimated 5-year OS, disease-specific survival and PFS rates were 79%, 80% and 74%, respectively. The main differences between the discovery and validation cohort was that the patients in the discovery cohort were younger and had clinically higher risk disease (Table 1).

### Identification of splicing events

**Figure 1:**
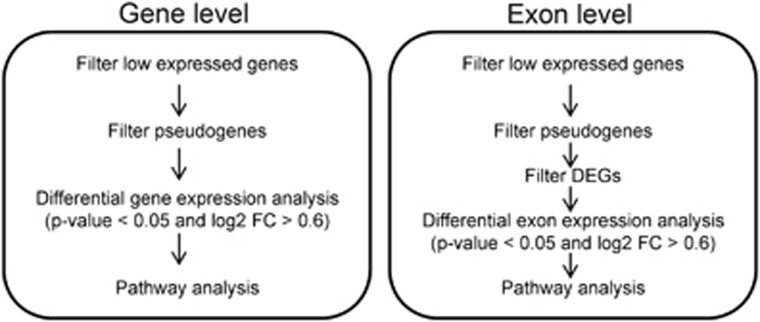
Flowchart: identification of differentially expressed genes in DLBCL.

Global mRNA and AS variations with prognostic impact were identified by comparing gene and exon expression profiles between the patients who experienced relapse (*n*=9, poor prognosis group) or remained in long-term remission (>24 months;^29^ *n*=29, good prognosis group) after chemoimmunotherapy (Figure 1). No significant differences were observed in baseline characteristics between the groups (Table 2). Using the gene-level expression data, 220 differentially expressed genes (DEGs) between the groups were identified (Supplementary Table S1). Of these, 59% were suppressed and 41% upregulated in patients with poor prognosis. The most upregulated genes included not only many nonprotein coding genes such as microRNAs and long noncoding RNAs but also *MME*, which is coding for CD10 and is included in the COO classification,^24^ and *TRAF4*, which has been shown to downregulate the innate immunity signaling.^30^ Innate immunity was also affected because of suppression of other genes in the pathway such as *HLA-DQB1*, and especially genes involved in T cell-mediated immune responses (*FOS*, *IL2RB*) or B-cell maturation (*IL7* and *KLF3*). The pathways significantly enriched among the DEGs included immune response-related processes, such as antigen processing and presentation, JAK/STAT (Janus kinase/signal transducer and activator of transcription) signaling, circadian rhythm and hematopoietic cell lineage (Table 3 and Supplementary Table S2).

To identify AS events between the good and poor prognosis groups, we applied similar criteria to exon-level expression data with additional filtering of the DEGs from the gene-level analysis (Figure 1). As a result, 8785 unique exonic regions from 3888 genes with differential expression were found (Supplementary Table S3). Among the genes with DEEs were genes with known alternative isoforms associated with cancer, including *RUNX1*,^31^ *TERT*^32, 33^ and *VEGFA*,^34^ as well as *CD44* and *PTPRO* that have previously been shown to be alternatively spliced in DLBCL.^17, 19, 20, 35^ In the case of *CD44*, differential exon inclusion was seen particularly in the variable region of the gene (exons v2–v10), involved in variant isoforms of *CD44* that have been shown to substantiate to poor prognosis in DLBCL in the pre-rituximab era.^18, 21^ In the *PTPRO* gene, the DEE was localized in the catalytic domain region, present also in the lymphoid-predominant truncated isoform, shown to modulate SYK phosphorylation and B-cell receptor activity.^36^ Other genes with the most suppressed exon expression in the poor prognosis group included *EPN1* and *ARAP1*, which are involved in endocytosis, and *HLA-DQA1*, *CD4*, *EBI3*, *ICAM1* and *UBA5*, which are related to the immune system. Differential splicing was also observed in many splicing factors and regulators, such as *NOVA1*, *TRA2B*, *SF3B1*, *ESRP1*, *RBFOX1* and *CELF5*, suggesting that the splicing mechanism might be affected through modulation of splicing factors themselves. In line with the pathways significantly enriched among the DEGs, the AS genes were significantly enriched for immune response-related pathways, such as antigen processing and presentation, and leukocyte transendothelial migration. In addition, the AS genes were enriched for the pathways related to adhesion, extracellular matrix interactions and signal transduction such as phosphatidylinositol signaling system and adenosine triphosphate (ATP)-binding cassette transporters (Table 3).

**Figure 2:**
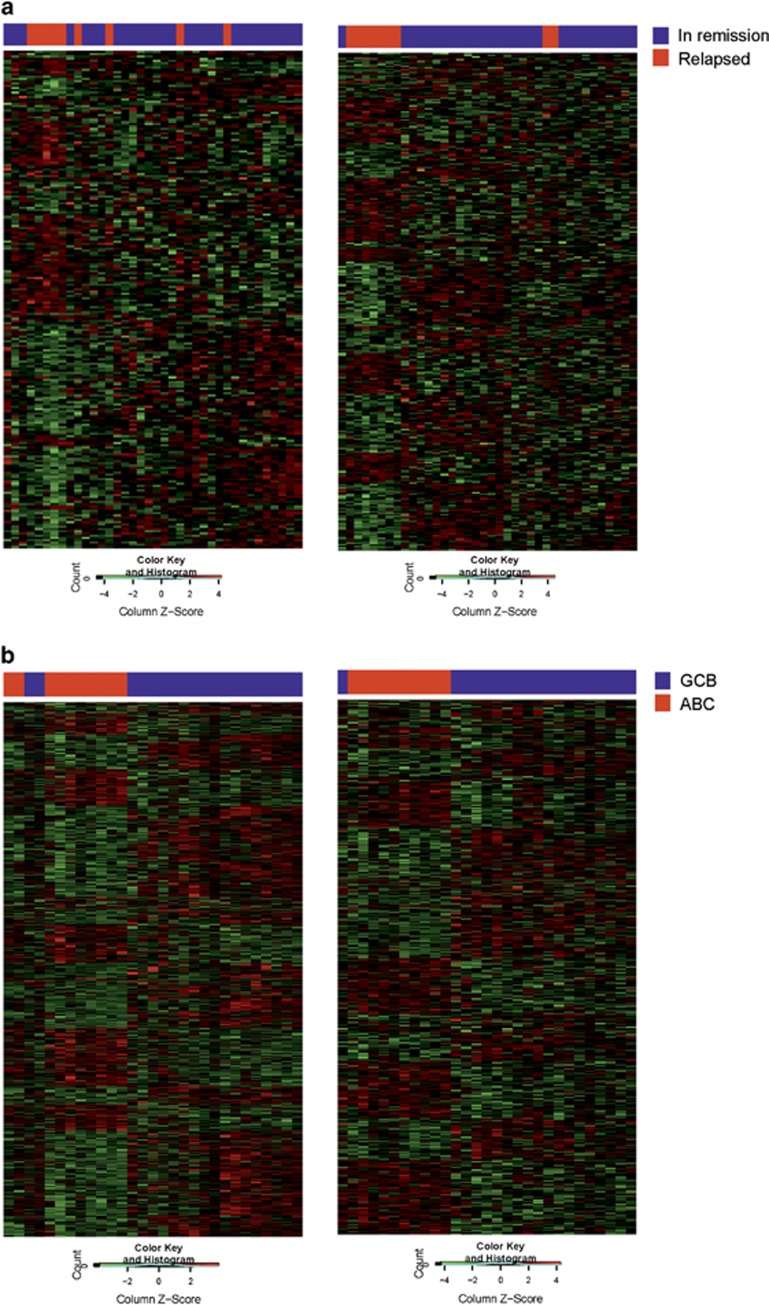
Clustering of the patients according to differentially expressed genes or exons. (**a**) A total of 220 DEGs and 315 DEEs were used for clustering the DLBCL patients who relapsed or patients who remained in remission. (**b**) A total of 1045 DEGs and 590 DEEs were used for clustering the ABC and GCB subgroups.

Interestingly, when the patient groups with poor and favorable outcomes were clustered according to the DEGs and DEEs, the expression profile of the most variable exons showed better separation of the groups than the profile of the most variable genes (Figure 2a). This indicates that AS plays an important role in DLBCL pathogenesis.

### Gene- and exon-level profiles according to COO subtypes

The observed differences in gene and exon levels between the patient groups with different outcomes raised the question of whether a similar phenomenon occurs when the patients are divided according to their molecular subtypes. When the gene expression-based COO classification was performed using previously described gene predictor,^24^ 19 of the cases were predicted as GCB, 12 as ABC and 7 as other DLBCL subtypes (Supplementary Figure S1). In this cohort, no significant association between the molecular subtypes and survival was found (not shown). With similar criteria as previously used in the screen for the outcome-related DEGs and genes with DEEs, 1012 unique DEGs (Supplementary Table S4) and 20 386 exonic regions from 6726 unique genes (Supplementary Table S5) were discovered to be differentially expressed between the GCB and the ABC DLBCLs. As expected, the DEGs included many of the genes used in the ‘Wright’ subtype classification (*SERPINA9*, *MYBL1*, *FUT8*, *IRF4*), as well as other genes (*FOXP1*, *MAPK10*) shown to have subtype preferential expression profile.^37, 38^ On the contrary, the DEEs corresponded for only two genes (*CCND2*, *DDB1*) used in the subtype classification. In the pathway analysis, the genes from differential gene expression and exon usage between GCB and ABC subtypes were enriched in, for instance, phosphatidylinositol signaling system, regulation of actin cytoskeleton, adherens junction, focal adhesion and pathways in cancer (Supplementary Table S6). Altogether, the number of DEGs and DEEs was higher in the COO classification than in the survival-related profiles. Clustering of the patient groups with molecular subtypes according to the DEGs or DEEs was able to separate the ABC and GCB subtypes (Figure 2b).

### Functional relevance of the alternative splicing

**Figure 3:**
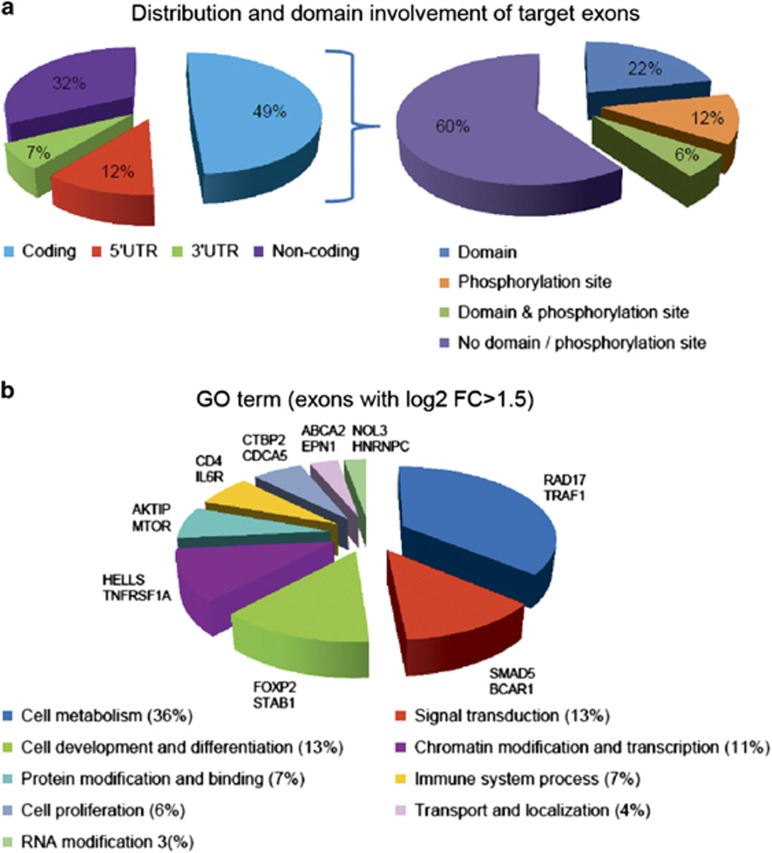
Distribution of the splicing events and characteristics of the target exons. (**a**) Distribution and domain involvement of the target exons. (**b**) Relative distribution of the target exons by Gene Ontology (GO) categories. Representative genes from each group are presented next to the pie chart.

To analyze in more detail the functional effect of the DEEs identified from the comparison of the groups with poor and favorable outcomes, we studied the association of the exonic regions with their corresponding protein domains using our exon-specific functional analysis pipeline. Altogether, 49% (4351) of the AS-related exons were protein coding, whereas 12% were mapped to 5′ UTR, 7% to the 3′ UTR and 32% to other noncoding regions (Figure 3a and Supplementary Table S7). Domain prediction showed 28% of translated exons (1193) to include a functional protein domain. In addition, 18% of the coding exons were predicted to include phosphorylation sites, of which 53% were serines, 25% treonines and 22% tyrosines (not shown).

In order to define the biological functions potentially affected by AS, we performed GO annotations for biological processes with the genes with most differentially expressed exons (log2 fold change ⩾∣1.5∣, *n*=289). To reduce the redundancy among several categories, related ontologies were regrouped into larger groups (Figure 3b). From the separate ontologies, the highest number of genes was annotated to signal transduction (for example, *SMAD5* and *BCAR1*). After regrouping, different cellular metabolic processes formed the most commonly implicated processes (for example, *RAD17* and *TRAF1*). Transport and localization annotation included, for example, *EPN1* that is involved in receptor-mediated endocytosis^39^ and ATP-binding cassette transporters *ABCA2*, *ABCA3* and *ABCA7* that are mostly known for their role in evolving drug resistance.^40^ Closer examination of ATP-binding cassette transporters revealed that many of the affected exons included a predicted functional protein domain or phosphorylation sites (not shown), suggesting that AS events might affect drug resistance by regulating the function and activity of the ATP-binding cassette transporters.

### Validation of exon inclusion events

To validate the outcome-associated DEEs, we used data from a publicly available CGCI (RNAseq cohort of 92 patients).^8^ Of the 3888 AS genes, 547 were validated in the CGCI cohort. Thirty-seven DEEs (33 unique genes), corresponding to 17 protein coding transcripts, were exactly in the same location with the expression difference in the same direction (Supplementary Table S8). According to Cox univariate analysis, 29 out of the 37 DEEs were associated with PFS (*P*⩽0.05) and 20 with OS in the CGCI cohort (Table 4). In Cox multivariate analyses with International Prognostic Index score, 22 DEEs retained independence for prediction of PFS (Supplementary Table S9). These included several interesting genes, such as *BCAR1* (coding for p130Cas, a docking protein involved in many intracellular signaling pathways), *APH1A* (*anterior pharynx defective-1α*, a component of the γ-secretase complex that cleaves integral membrane proteins such as Notch receptors and β-amyloid precursor protein), *KCNH6* (member of a potassium voltage-gated channel family, shown to be associated with drug sensitivity) and *CUL3* (*cullin 3*, a core component of an E3 ubiquitin ligase complex).

**Figure 4:**
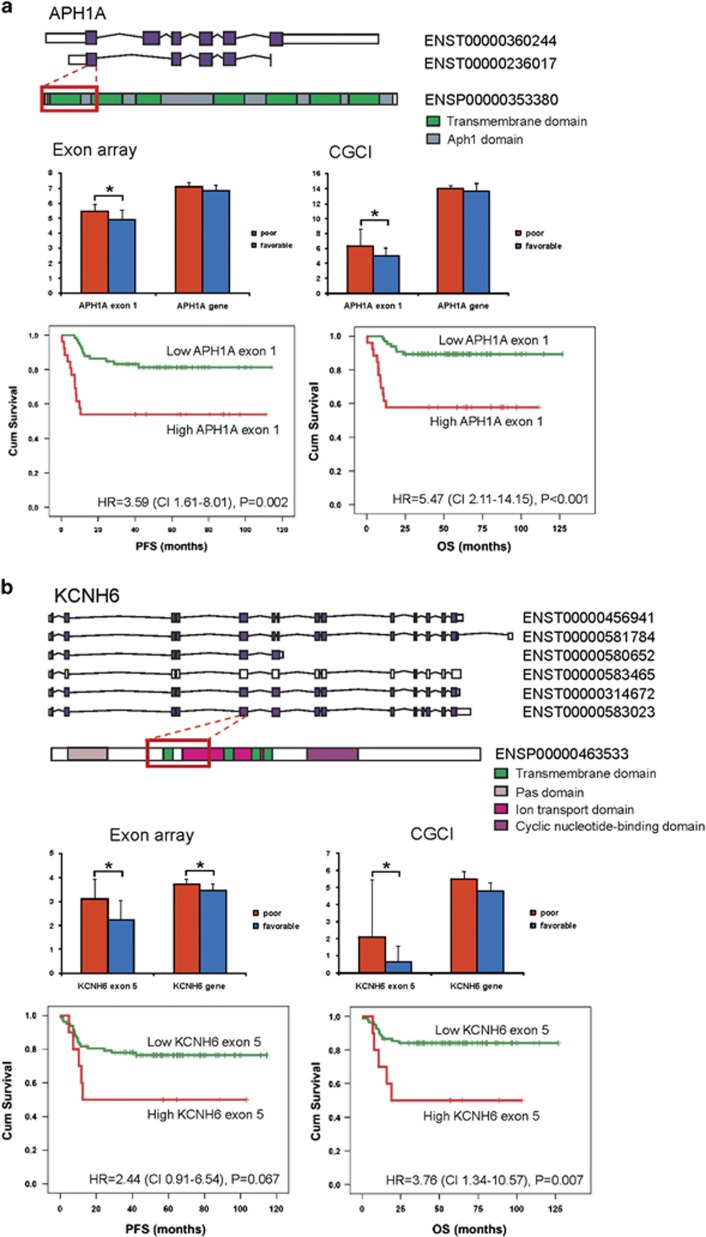

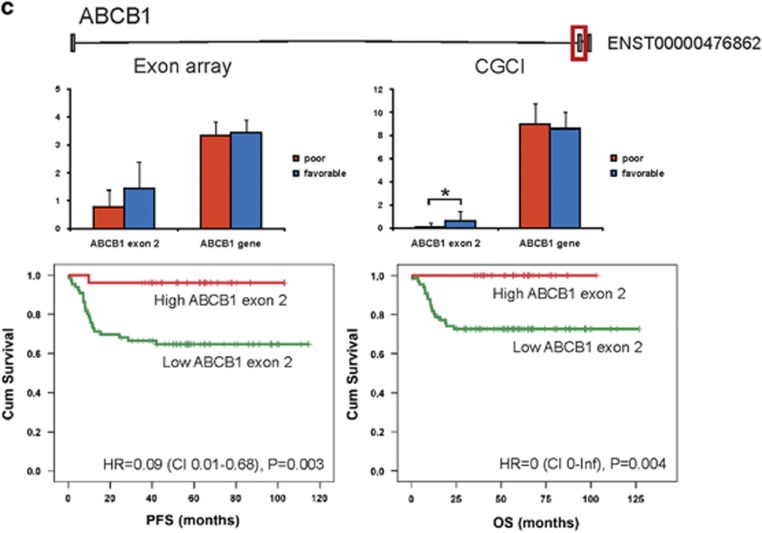
Differentially expressed exons may affect the functional properties of the protein and are associated with survival. (**a**–**c**) The upper panels show the domain information, the middle panels show the exon and gene expression in the discovery and validation cohorts and the lower panels show Kaplan–Meier survival plots of the exons in DLBCL patients (validation cohort).

Some of the validated survival-associated DEEs that targeted protein coding regions could be linked to their protein-level functions. For example, the first coding exon of *APH1A* overexpressed in patients with poor prognosis corresponded to the entire first APH-1 transmembrane domain (Figure 4a). Similarly, the exon with differential expression in the *KCNH6* gene mapped to the amino acids 226–367 including three transmembrane helix regions, and exon skipping was associated with better survival (Figure 4b). This suggests that differential coding exon usage could alter the protein functions and thus result in drug resistance and disease progression. On the contrary, *ABCB1* represents a gene, where exon 2 skipping occurring in the promoter region is an unfavorable event and correlates with poor survival (Figure 4c).

## Discussion

There has been an increasing interest in studying whole genome-level alterations in DLBCL during the recent years.^9^ The contribution of these studies in the identification of key elements in DLBCL pathogenesis has been crucial, yet the impact of AS on DLBCL pathogenesis and survival has to date remained largely unexplored. In this work, we have performed microarray-based exon and whole transcriptome profiling on freshly frozen lymphoma tissue collected prospectively from DLBCL patients treated homogenously in a Nordic phase II study.^22^ We show association of AS genes with molecular subtypes and survival. Interestingly, exon-level profile can separate clinically high-risk DLBCL patients into subgroups with poor and favorable survival more accurately than gene-level profile. We observe that many of the genes are differentially expressed only at the exon level but not at the gene level and would have been missed if only gene-level analysis or conventional 3′ oligonucleotide microarrays had been used. Finally, we validate molecular subtype and survival association of the key DEGs and DEEs in an independent DLBCL cohort. Our observations provide important basis for understanding the mechanisms of DLBCL pathogenesis and prognosis, and development of novel therapeutic strategies.

Approximately half of the AS events target noncoding regions. A significant proportion of the alterations affected 5′ and 3′ UTRs, suggesting modulation of epigenetic regulatory pathways in DLBCL. Although alternative exon usage in the coding regions can generate different protein isoforms, splicing in the noncoding 3′ UTRs can compromise microRNA-dependent gene regulation and change the composition of translational regulatory elements, or result in differential promoter usage in 5′ UTRs that in turn can further alter protein expression.^41^ Moreover, the upstream promoter site may be selectively affected and differential promoter usage produce N-terminal splice variants.^42^ Our results further suggest that the splicing mechanism might be affected through modulation of splicing factors themselves, providing yet another regulatory level for gene expression.

A novel finding from the pathway analysis of the DEGs was the enrichment of the genes in the circadian rhythm pathway. Disturbances of the mammalian clock genes have been linked to tumorigenesis. In DLBCL, circadian genes *CEBPA* and its downstream target *PER2* are highly deregulated,^43^ and *BMAL1* (*ARNTL*) is epigenetically inactivated.^44^ Furthermore, genetic variants of *CRY2* have been associated with a risk of non-Hodgkin’s lymphoma. In our exon array results, the expression of the circadian clock genes *PER1* and *ARNTL* was reduced in relapsed patients and thus associated with dismal prognosis.

ATP-binding cassette transporters, in turn, represent a novel group of genes we found to be regulated at the exon level and thereby potentially affected by AS. To date, ATP-binding cassette transporters have been mostly recognized from their contribution to drug resistance. The highly conserved ATP-binding cassette domains of these transporters provide the ATP-powered translocation of many substrates across the membranes, whereas the transmembrane domains creating the translocation pathway are more variable. According to our domain prediction analysis of splicing-associated ATP-binding cassette transporters, many of the affected domains included either a transmembrane domain or an ATP-binding cassette domain that might directly have an effect to the proper function of the transporter. Most of the AS-associated ATP-binding cassette transporters belonged to ABCA family. These included genes encoding *ABCA2* and *ABCA3* that have been shown to contribute to drug resistance in T-cell acute lymphoblastic leukemia.^45^ In addition, increased expression of ABCA3 has been linked to enhanced exosomal evasion of humoral immunotherapy.^46^

We validated the DEGs and DEEs in an independent patient cohort consisting of RNAseq data. *APH1A* was one of the interesting examples of the AS genes that could be validated by RNAseq and linked to protein-level functions. It encodes an essential component of the multi-transmembrane γ-secretase complex that is required for the cleavage and activation of integral membrane proteins, including Notch.^47^ Considering the increasing evidence of the deregulated Notch signaling in cancer progression, γ-secretases playing an important role in Notch activation and that *APH1A* is critically required for γ-secretase activity, our finding showing that alternative exon usage of *APH1A* gene has prognostic impact in DLBCL may have therapeutic implications. Thus, future studies should be directed to enhance our understanding of the function of *APH1A* isoforms and their relation to Notch signaling in DLBCL that may further improve opportunities for the design of selective lymphoma therapeutics.

*KCNH6*, also known as *HERG2*, encodes a pore-forming subunit of a voltage-gated potassium channel. Potassium channels comprise the largest family of ion channels encoded by genes with phenotypic diversity generated through alternative splicing, variable association of channel subunits and posttranslational modifications. Recent data indicate that blocking of the ion channel activity can impair cancer cell growth.^48^ Interestingly, HERG potassium channels are constitutively active in acute myeloid leukemia,^49, 50^ and have favorable prognostic impact on survival. In our study, no differences were observed in *HERG2* gene expression levels between poor and good prognosis patients. However, low expression of exon 5, which corresponds to the first transmembrane and ion transport domains, was associated with favorable survival. The result suggests that these functional domains mediate prosurvival signals that can be overcome by exon 5 skipping. *ABCB1* in turn represents a gene where exon 2 skipping occurring in the promoter region is an unfavorable event correlating with lymphoma progression.

Taken together, the results presented herein are promising and novel, and suggest a significant role for alternative exon usage in the molecular profiles of DLBCL. The importance of studying connections between splicing and DLBCL is emphasized by the possibility that some AS isoforms drive progression and may represent attractive therapeutic targets even if total gene expression is not affected. Moreover, unique or sets of isoforms may be used as biomarkers for disease progression.

## Figures and Tables

**Table 1:** Patient characteristics of the discovery and validation cohorts

*Number of patients*	*Discovery cohort,* n *(%)*	*Validation cohort,* n *(%)*
Total	38 (100)	92 (100)
		
*Gender*		
Female	14 (37)	31 (34)
Male	24 (63)	61 (66)
		
*Age*		
<60	28 (74)	39 (42)
60–65	10 (26)	15 (16)
>65	0 (0)	38 (41)
*Subtype*		
GCB	19 (50)	51 (55)
ABC	12 (32)	32 (35)
Other/unclassified	7 (18)	9 (10)
		
*Performance status*		
0–1	24 (63)	64 (70)
2–3	14 (37)	28 (30)
B-symptoms	22 (58)	
Bulky disease	22 (58)	
Elevated LDH	37 (97)	
		
*Stage*		
I–II	1 (3)	44 (48)
III–IV	37 (97)	48 (52)
		
*IPI*		
0	0 (0)	16 (17)
1	0 (0)	24 (26)
2	17 (45)	22 (24)
3	10 (26)	18 (20)
4	10 (26)	10 (11)
5	1 (3)	2 (2)

Abbreviations: ABC, activated B-cell; GCB, germinal center B-cell; IPI, International Prognostic Index; LDH, lactate dehydrogenase.

**Table 2:** Baseline characteristics of the discovery cohort according to good and poor prognosis

*Patients*	*Good prognosis,* n *(%)*	*Poor prognosis,* n *(%)*	P*-value*
Total	29 (76)	9 (24)	
			
*Gender*			
Female	19 (66)	5 (56)	0.699
Male	10 (34)	4 (44)	
			
*Age*			
<60	23 (79)	6 (67)	0.655
>60	6 (21)	3 (33)	
			
*Subtype*			
GCB	17 (59)	7 (88)	0.438
Non-GCB	12 (41)	2 (22)	
			
*Performance status*			
0–1	20 (69)	4 (44)	0.245
2–3	9 (31)	5 (56)	
			
*B-symptoms*			
No	10 (34)	6 (67)	0.128
Yes	19 (66)	3 (33)	
			
*Bulky disease*			
No	13 (45)	3 (33)	0.706
Yes	16 (55)	6 (67)	
			
*Elevated LDH*			
No	0 (0)	1 (11)	0.237
Yes	29 (100)	8 (89)	
			
*Stage*			
I–II	1 (3)	0 (0)	1.000
III–IV	28 (97)	9 (100)	
			
*aaIPI*			
3–4	21 (72)	6 (67)	1.000
	8 (28)	3 (33)	
			
*BCL2*			
Low	27 (93)	7 (78)	0.233
High^a^	2 (7)	2 (22)	
			
*cMYC*			
Low	28 (97)	9 (100)	1.000
High	1 (3)	0 (0)	

Abbreviations: aaIPI, age-adjusted International Prognostic Index; GCB, germinal center B-cell; LDH, lactate dehydrogenase.

a *Z*-score >2.0.

**Table 3:** Pathways significantly enriched among the DEGs and DEEs between good and poor prognosis groups

*Pathway name*	*Input/total no. of genes*	*FDR*
*Gene level*		
Antigen processing and presentation	2/89	<0.001
Circadian rhythm	2/13	<0.001
JAK/STAT signaling pathway	7/155	0.001
Hematopoietic cell lineage	5/87	0.003
Biosynthesis of unsaturated fatty acids	3/22	0.004
		
*Exon level (top 20 pathways)*		
Antigen processing and presentation	17/89	<0.001
Leukocyte transendothelial migration	22/119	<0.001
Cell adhesion molecules (CAMs)	26/134	<0.001
Adherens junction	19/78	<0.001
Phosphatidylinositol signaling system	21/76	<0.001
ECM receptor interaction	33/84	<0.001
Focal adhesion	59/203	<0.001
Pathways in cancer	84/330	<0.001
Calcium signaling pathway	50/182	<0.001
ABC transporters	19/44	<0.001
MAPK signaling pathway	64/272	<0.001
Long-term depression	25/75	<0.001
Tight junction	36/135	<0.001
Amyotrophic lateral sclerosis (ALS)	19/56	0.001
Small cell lung cancer	25/86	0.001
VEGF signaling pathway	22/74	0.002
Axon guidance	33/129	0.002
Type I diabetes mellitus	14/44	0.002
Regulation of actin cytoskeleton	47/217	0.003
Thyroid cancer	11/29	0.003

Abbreviations: ABC, adenosine triphosphate-binding cassette; ECM, extracellular matrix; DEE, differentially expressed exon; DEG, differentially expressed gene; FDR, false discovery rate; JAK/STAT, Janus kinase/signal transducer and activator of transcription; MAPK, mitogen-activated protein kinase; VEGF, vascular endothelial growth factor.

**Table 4:** Cox univariate analysis of the DEEs common in the discovery and validation cohorts (significant *P*<0.05 are in bold)

*Gene*	*Discovery cohort*				*Validation cohort*			
	*PFS*		*OS*		*PFS*		*OS*	
	*P-val exon*	*P-val gene*	*P-val exon*	*P-val gene*	*P-val exon*	*P-val gene*	*P-val exon*	*P-val gene*
*CUL3*	0.070	0.975	0.167	0.728	**0.007**	0.393	**0.024**	0.399
*DKK3*	0.091	0.330	0.099	0.427	**0.037**	0.497	0.064	0.623
*BCAR1*	**0.022**	0.179	0.108	0.499	**0.005**	0.439	**0.002**	0.449
*SLC9A3*	0.112	0.154	0.084	**0.016**	**0.002**	0.164	**0.002**	0.139
*GAL*	0.051	0.938	0.468	0.475	**0.003**	0.687	**0.033**	0.659
*ABCB1*	0.116	0.525	0.204	0.806	**0.019**	0.343	**0.045**	0.895
*NAMPT*	**0.016**	0.757	0.138	0.401	**0.015**	**0.003**	0.121	**0.004**
*CUBN*	**0.035**	0.285	**0.038**	0.246	**0.047**	**0.024**	0.079	**0.018**
*APH1A*	**0.048**	0.123	**0.039**	0.043	**<0.001**	**0.011**	**<0.001**	**0.016**
*SSUH2*	**0.010**	0.201	**0.015**	0.113	**0.022**	0.396	0.400	0.094
*RHOT1*	0.099	0.597	0.126	0.610	**0.001**	**0.023**	**0.002**	**0.027**
*NHSL1*	**0.012**	0.732	**0.037**	0.700	**<0.001**	0.153	**0.007**	0.354
*TACC2*	0.091	0.973	0.177	0.728	**0.002**	0.183	0.087	0.245
*FRAS1*	**0.002**	**0.039**	**0.001**	0.051	**0.001**	0.107	**0.033**	0.085
*PTPRQ*	**0.013**	0.862	0.170	0.861	0.130	0.404	**0.018**	**0.032**
*CYP4B1*	0.051	0.327	0.054	0.150	**0.013**	0.269	0.136	0.268
*HMGCLL1*	**0.030**	0.060	0.597	0.299	**0.011**	0.834	**0.023**	0.395
*FAM83A*	**0.018**	0.207	**0.002**	0.348	**0.003**	0.865	**0.029**	0.671
*CPB1*	**0.020**	0.330	**0.024**	0.228	**0.006**	0.394	**0.002**	0.185
*SORBS2*	0.104	0.418	0.285	0.962	0.101	0.151	0.417	0.149
*C2orf65*	**0.042**	0.273	0.361	0.583	**<0.001**	0.238	**0.010**	0.173
*CAMK2N1*	**0.011**	0.433	**0.009**	0.794	0.489	**0.028**	0.986	0.094
*NOS3*	**0.022**	0.246	**0.031**	0.687	**0.034**	0.753	0.064	0.760
*KCNH6*	**0.015**	**0.019**	0.080	**0.008**	**0.001**	**0.050**	**0.001**	**0.002**
*TRIML2*	**0.005**	**0.005**	**0.008**	**0.003**	**0.015**	0.338	**0.019**	0.859
*MAP3K15*	**0.011**	**0.024**	0.142	**0.025**	**0.001**	0.578	**0.003**	0.217
*TMEM232*	**0.030**	0.359	0.172	0.298	**0.001**	**0.034**	**0.001**	**0.003**
*CYHR1*	**0.041**	0.167	0.086	**0.019**	**0.001**	0.724	**0.001**	0.586
*VEPH1*	0.125	0.092	0.282	**0.04**	**<0.001**	**0.025**	**0.006**	0.062
*AC018705.5*	**0.013**	0.061	**0.012**	**0.045**	**0.018**	0.920	0.078	0.876
*SPANXA2_OT1*	**0.029**	**0.048**	0.151	0.076	0.124	0.053	0.153	0.200
*RP11_69C17.1*	**0.007**	0.400	**0.029**	0.636	**0.012**	0.246	0.131	0.561
*RP11_696N14.1*	0.068	0.160	0.614	0.447	**0.017**	0.982	0.190	0.691

Abbreviations: DEE, differentially expressed exon; OS, overall survival; PFS, progression-free survival.

## References

[bib1] Coiffier B, Lepage E, Briere J, Herbrecht R, Tilly H, Bouabdallah R et al. CHOP chemotherapy plus rituximab compared with CHOP alone in elderly patients with diffuse large-B-cell lymphoma. N Engl J Med 2002; 346: 235–242. 11807147 10.1056/NEJMoa011795

[bib2] Pfreundschuh M, Trumper L, Osterborg A, Pettengell R, Trneny M, Imrie K et al. CHOP-like chemotherapy plus rituximab versus CHOP-like chemotherapy alone in young patients with good-prognosis diffuse large-B-cell lymphoma: a randomised controlled trial by the MabThera International Trial (MInT) Group. Lancet Oncol 2006; 7: 379–391. 16648042 10.1016/S1470-2045(06)70664-7

[bib3] Alizadeh AA, Eisen MB, Davis RE, Ma C, Lossos IS, Rosenwald A et al. Distinct types of diffuse large B-cell lymphoma identified by gene expression profiling. Nature 2000; 403: 503–511. 10676951 10.1038/35000501

[bib4] Lenz G, Wright G, Dave SS, Xiao W, Powell J, Zhao H et al. Stromal gene signatures in large-B-cell lymphomas. N Engl J Med 2008; 359: 2313–2323. 19038878 10.1056/NEJMoa0802885 PMC9103713

[bib5] Rosenwald A, Wright G, Chan WC, Connors JM, Campo E, Fisher RI et al. The use of molecular profiling to predict survival after chemotherapy for diffuse large-B-cell lymphoma. N Engl J Med 2002; 346: 1937–1947. 12075054 10.1056/NEJMoa012914

[bib6] Rosenwald A, Wright G, Leroy K, Yu X, Gaulard P, Gascoyne RD et al. Molecular diagnosis of primary mediastinal B cell lymphoma identifies a clinically favorable subgroup of diffuse large B cell lymphoma related to Hodgkin lymphoma. J Exp Med 2003; 198: 851–862. 12975453 10.1084/jem.20031074 PMC2194208

[bib7] Morin RD, Mendez-Lago M, Mungall AJ, Goya R, Mungall KL, Corbett RD et al. Frequent mutation of histone-modifying genes in non-Hodgkin lymphoma. Nature 2011; 476: 298–303. 21796119 10.1038/nature10351 PMC3210554

[bib8] Morin RD, Mungall K, Pleasance E, Mungall AJ, Goya R, Huff RD et al. Mutational and structural analysis of diffuse large B-cell lymphoma using whole-genome sequencing. Blood 2013; 122: 1256–1265. 23699601 10.1182/blood-2013-02-483727 PMC3744992

[bib9] Pasqualucci L, Dalla-Favera R. The genetic landscape of diffuse large B-cell lymphoma. Semin Hematol 2015; 52: 67–76. 25805586 10.1053/j.seminhematol.2015.01.005 PMC4646421

[bib10] Pan Q, Shai O, Lee LJ, Frey BJ, Blencowe BJ. Deep surveying of alternative splicing complexity in the human transcriptome by high-throughput sequencing. Nat Genet 2008; 40: 1413–1415. 18978789 10.1038/ng.259

[bib11] Chen J, Weiss WA. Alternative splicing in cancer: implications for biology and therapy. Oncogene 2015; 34: 1–14. 24441040 10.1038/onc.2013.570

[bib12] Pajares MJ, Ezponda T, Catena R, Calvo A, Pio R, Montuenga LM. Alternative splicing: an emerging topic in molecular and clinical oncology. Lancet Oncol 2007; 8: 349–357. 17395108 10.1016/S1470-2045(07)70104-3

[bib13] Venables JP. Unbalanced alternative splicing and its significance in cancer. Bioessays 2006; 28: 378–386. 16547952 10.1002/bies.20390

[bib14] Zhang C, Li HR, Fan JB, Wang-Rodriguez J, Downs T, Fu XD et al. Profiling alternatively spliced mRNA isoforms for prostate cancer classification. BMC Bioinformatics 2006; 7: 202. 16608523 10.1186/1471-2105-7-202 PMC1458362

[bib15] Zhang Z, Pal S, Bi Y, Tchou J, Davuluri RV. Isoform level expression profiles provide better cancer signatures than gene level expression profiles. Genome Med 2013; 5: 33. 23594586 10.1186/gm437 PMC3706752

[bib16] Brown PJ, Ashe SL, Leich E, Burek C, Barrans S, Fenton JA et al. Potentially oncogenic B-cell activation-induced smaller isoforms of FOXP1 are highly expressed in the activated B cell-like subtype of DLBCL. Blood 2008; 111: 2816–2824. 18077790 10.1182/blood-2007-09-115113

[bib17] Aguiar RC, Yakushijin Y, Kharbanda S, Tiwari S, Freeman GJ, Shipp MA. PTPROt: an alternatively spliced and developmentally regulated B-lymphoid phosphatase that promotes G0/G1 arrest. Blood 1999; 94: 2403–2413. 10498613

[bib18] Nagel S, Hirschmann P, Dirnhofer S, Gunthert U, Tzankov A. Coexpression of CD44 variant isoforms and receptor for hyaluronic acid-mediated motility (RHAMM, CD168) is an International Prognostic Index and C-MYC gene status-independent predictor of poor outcome in diffuse large B-cell lymphomas. Exp Hematol 2010; 38: 38–45. 19857547 10.1016/j.exphem.2009.10.010

[bib19] Stauder R, Eisterer W, Thaler J, Gunthert U. CD44 variant isoforms in non-Hodgkin's lymphoma: a new independent prognostic factor. Blood 1995; 85: 2885–2899. 7537983

[bib20] Terpe HJ, Koopmann R, Imhof BA, Gunthert U. Expression of integrins and CD44 isoforms in non-Hodgkin's lymphomas: CD44 variant isoforms are preferentially expressed in high-grade malignant lymphomas. J Pathol 1994; 174: 89–100. 7525912 10.1002/path.1711740205

[bib21] Tzankov A, Pehrs AC, Zimpfer A, Ascani S, Lugli A, Pileri S et al. Prognostic significance of CD44 expression in diffuse large B cell lymphoma of activated and germinal centre B cell-like types: a tissue microarray analysis of 90 cases. J Clin Pathol 2003; 56: 747–752. 14514777 10.1136/jcp.56.10.747 PMC1770073

[bib22] Holte H, Leppa S, Bjorkholm M, Fluge O, Jyrkkio S, Delabie J et al. Dose-densified chemoimmunotherapy followed by systemic central nervous system prophylaxis for younger high-risk diffuse large B-cell/follicular grade 3 lymphoma patients: results of a phase II Nordic Lymphoma Group study. Ann Oncol 2013; 24: 1385–1392. 23247661 10.1093/annonc/mds621

[bib23] Chen P, Lepikhova T, Hu Y, Monni O, Hautaniemi S. Comprehensive exon array data processing method for quantitative analysis of alternative spliced variants. Nucleic Acids Res 2011; 39: e123. 21745820 10.1093/nar/gkr513 PMC3185423

[bib24] Wright G, Tan B, Rosenwald A, Hurt EH, Wiestner A, Staudt LM. A gene expression-based method to diagnose clinically distinct subgroups of diffuse large B cell lymphoma. Proc Natl Acad Sci USA 2003; 100: 9991–9996. 12900505 10.1073/pnas.1732008100 PMC187912

[bib25] Subramanian A, Tamayo P, Mootha VK, Mukherjee S, Ebert BL, Gillette MA et al. Gene set enrichment analysis: a knowledge-based approach for interpreting genome-wide expression profiles. Proc Natl Acad Sci USA 2005; 102: 15545–15550. 16199517 10.1073/pnas.0506580102 PMC1239896

[bib26] Budczies J, Klauschen F, Sinn BV, Gyorffy B, Schmitt WD, Darb-Esfahani S et al. Cutoff Finder: a comprehensive and straightforward Web application enabling rapid biomarker cutoff optimization. PLoS ONE 2012; 7: e51862. 23251644 10.1371/journal.pone.0051862 PMC3522617

[bib27] Jones P, Binns D, Chang HY, Fraser M, Li W, McAnulla C et al. InterProScan 5: genome-scale protein function classification. Bioinformatics 2014; 30: 1236–1240. 24451626 10.1093/bioinformatics/btu031 PMC3998142

[bib28] Hornbeck PV, Kornhauser JM, Tkachev S, Zhang B, Skrzypek E, Murray B et al. PhosphoSitePlus: a comprehensive resource for investigating the structure and function of experimentally determined post-translational modifications in man and mouse. Nucleic Acids Res 2012; 40(Database issue): D261–D270. 22135298 10.1093/nar/gkr1122 PMC3245126

[bib29] Maurer MJ, Ghesquieres H, Jais JP, Witzig TE, Haioun C, Thompson CA et al. Event-free survival at 24 months is a robust end point for disease-related outcome in diffuse large B-cell lymphoma treated with immunochemotherapy. J Clin Oncol 2014; 32: 1066–1073. 24550425 10.1200/JCO.2013.51.5866 PMC3965261

[bib30] Marinis JM, Hutti JE, Homer CR, Cobb BA, Cantley LC, McDonald C et al. IkappaB kinase alpha phosphorylation of TRAF4 downregulates innate immune signaling. Mol Cell Biol 2012; 32: 2479–2489. 22547678 10.1128/MCB.00106-12 PMC3434482

[bib31] Gattenloehner S, Chuvpilo S, Langebrake C, Reinhardt D, Muller-Hermelink HK, Serfling E et al. Novel RUNX1 isoforms determine the fate of acute myeloid leukemia cells by controlling CD56 expression. Blood 2007; 110: 2027–2033. 17431130 10.1182/blood-2007-02-074203

[bib32] Amor S, Remy S, Dambrine G, Le Vern Y, Rasschaert D, Laurent S. Alternative splicing and nonsense-mediated decay regulate telomerase reverse transcriptase (TERT) expression during virus-induced lymphomagenesis *in vivo*. BMC Cancer 2010; 10: 571. 20964812 10.1186/1471-2407-10-571 PMC2976754

[bib33] Wang Y, Meeker AK, Kowalski J, Tsai HL, Somervell H, Heaphy C et al. Telomere length is related to alternative splice patterns of telomerase in thyroid tumors. Am J Pathol 2011; 179: 1415–1424. 21763260 10.1016/j.ajpath.2011.05.056 PMC3157225

[bib34] Biselli-Chicote PM, Oliveira AR, Pavarino EC, Goloni-Bertollo EM. VEGF gene alternative splicing: pro- and anti-angiogenic isoforms in cancer. J Cancer Res Clin Oncol 2012; 138: 363–370. 22045472 10.1007/s00432-011-1073-2 PMC11824452

[bib35] Akisik E, Bavbek S, Dalay N. CD44 variant exons in leukemia and lymphoma. Pathol Oncol Res 2002; 8: 36–40. 11994761 10.1007/BF03033699

[bib36] Chen L, Juszczynski P, Takeyama K, Aguiar RC, Shipp MA. Protein tyrosine phosphatase receptor-type O truncated (PTPROt) regulates SYK phosphorylation, proximal B-cell-receptor signaling, and cellular proliferation. Blood 2006; 108: 3428–3433. 16888096 10.1182/blood-2006-03-013821

[bib37] Barrans SL, Fenton JA, Banham A, Owen RG, Jack AS. Strong expression of FOXP1 identifies a distinct subset of diffuse large B-cell lymphoma (DLBCL) patients with poor outcome. Blood 2004; 104: 2933–2935. 15238418 10.1182/blood-2004-03-1209

[bib38] Blenk S, Engelmann J, Weniger M, Schultz J, Dittrich M, Rosenwald A et al. Germinal center B cell-like (GCB) and activated B cell-like (ABC) type of diffuse large B cell lymphoma (DLBCL): analysis of molecular predictors, signatures, cell cycle state and patient survival. Cancer Inform 2007; 3: 399–420. 19455257 PMC2675856

[bib39] Chen H, Fre S, Slepnev VI, Capua MR, Takei K, Butler MH et al. Epsin is an EH-domain-binding protein implicated in clathrin-mediated endocytosis. Nature 1998; 394: 793–797. 9723620 10.1038/29555

[bib40] Tamaki A, Ierano C, Szakacs G, Robey RW, Bates SE. The controversial role of ABC transporters in clinical oncology. Essays Biochem 2011; 50: 209–232. 21967059 10.1042/bse0500209 PMC6944313

[bib41] Diederichs S, Bartsch L, Berkmann JC, Frose K, Heitmann J, Hoppe C et al. The dark matter of the cancer genome: aberrations in regulatory elements, untranslated regions, splice sites, non-coding RNA and synonymous mutations. EMBO Mol Med 2016; 8: 442–457. 26992833 10.15252/emmm.201506055 PMC5126213

[bib42] Zavolan M, Kondo S, Schonbach C, Adachi J, Hume DA, Hayashizaki Y et al. Impact of alternative initiation, splicing, and termination on the diversity of the mRNA transcripts encoded by the mouse transcriptome. Genome Res 2003; 13: 1290–1300. 12819126 10.1101/gr.1017303 PMC403716

[bib43] Thoennissen NH, Thoennissen GB, Abbassi S, Nabavi-Nouis S, Sauer T, Doan NB et al. Transcription factor CCAAT/enhancer-binding protein alpha and critical circadian clock downstream target gene PER2 are highly deregulated in diffuse large B-cell lymphoma. Leuk Lymphoma 2012; 53: 1577–1585. 22260161 10.3109/10428194.2012.658792 PMC3864038

[bib44] Taniguchi H, Fernandez AF, Setien F, Ropero S, Ballestar E, Villanueva A et al. Epigenetic inactivation of the circadian clock gene BMAL1 in hematologic malignancies. Cancer Res 2009; 69: 8447–8454. 19861541 10.1158/0008-5472.CAN-09-0551

[bib45] Efferth T, Gillet JP, Sauerbrey A, Zintl F, Bertholet V, de Longueville F et al. Expression profiling of ATP-binding cassette transporters in childhood T-cell acute lymphoblastic leukemia. Mol Cancer Ther 2006; 5: 1986–1994. 16928819 10.1158/1535-7163.MCT-06-0086

[bib46] Aung T, Chapuy B, Vogel D, Wenzel D, Oppermann M, Lahmann M et al. Exosomal evasion of humoral immunotherapy in aggressive B-cell lymphoma modulated by ATP-binding cassette transporter A3. Proc Natl Acad Sci USA 2011; 108: 15336–15341. 21873242 10.1073/pnas.1102855108 PMC3174603

[bib47] Lee SF, Shah S, Li H, Yu C, Han W, Yu G. Mammalian APH-1 interacts with presenilin and nicastrin and is required for intramembrane proteolysis of amyloid-beta precursor protein and Notch. J Biol Chem 2002; 277: 45013–45019. 12297508 10.1074/jbc.M208164200

[bib48] Jehle J, Schweizer PA, Katus HA, Thomas D. Novel roles for hERG K(+) channels in cell proliferation and apoptosis. Cell Death Dis 2011; 2: e193. 21850047 10.1038/cddis.2011.77 PMC3181422

[bib49] Pillozzi S, Masselli M, De Lorenzo E, Accordi B, Cilia E, Crociani O et al. Chemotherapy resistance in acute lymphoblastic leukemia requires hERG1 channels and is overcome by hERG1 blockers. Blood 2011; 117: 902–914. 21048156 10.1182/blood-2010-01-262691

[bib50] Smith GA, Tsui HW, Newell EW, Jiang X, Zhu XP, Tsui FW et al. Functional up-regulation of HERG K+ channels in neoplastic hematopoietic cells. J Biol Chem 2002; 277: 18528–18534. 11893742 10.1074/jbc.M200592200

