# Supplementary material for: Alternative splicing discriminates molecular subtypes and has prognostic impact in diffuse large B-cell lymphoma
Source: Blood Cancer J. 2017 Aug 25;7(8):e596–. doi: 10.1038/bcj.2017.71 (PMC5596382; doi:10.1038/bcj.2017.71)
Supplement: Supplementary Information [file bcj201771x1.docx]

**Supplementary Information**

**Alternative splicing discriminates molecular subtypes and has prognostic impact in diffuse large B-cell lymphoma**

Suvi-Katri Leivonen^1,2*^, Minna Taskinen^1,2*^, Alejandra Cervera^1^, Marja-Liisa Karjalainen-Lindsberg^3^, Jan Delabie^4**^, Harald Holte^5^, Rainer Lehtonen^1^, Sampsa Hautaniemi^1^ and Sirpa Leppä^1,2^

**Supplementary Materials and Methods**

**Patients**

The prospectively collected discovery cohort consisted of 38 DLBCL patients less than 65 years old with clinically high-risk (age adjusted International Prognostic Index (aaIPI) Score 2-3) disease. The patients were treated in a Nordic phase II NLG-LBC-04 protocol with six courses of R-CHOEP-14 (rituximab, cyclophosphamide, doxorubicin, vincristine, etoposide, and prednisone supported with G-CSF) followed by systemic CNS prophylaxis with one course of high-dose methotrexate and one course of high-dose cytarabine^1^. The original clinical study included 156 eligible patients. Histological diagnosis was established from surgical or needle biopsy of the pretreatment tumor tissue by local pathologists according to current criteria of the World Health Organization classification^2^, and subsequently reviewed by expert hematopathologists on a national basis. The patient selection of the study population was based on the availability of fresh frozen tissue containing adequate material for the analyses. The infiltration of lymphoma cells in the tissue was assessed from frozen tissue section with hematoxylin-eosin and toluidine blue stainings. All tissue samples were taken before treatments.

**Molecular subgroup prediction**

Samples from the 38 DLBCL patients with exon array data were classified into GCB, ABC and non-classified subgroups using the DLBCL subgroup gene predictor from Lymphochip data as previously described^3^. After excluding genes with median expression <32 across all samples, 21 out of the 27 genes in the DLBCL subgroup predictor were present on the Affymetrix Human Exon 1.0 ST array and passed the filtering step. These 21 genes were used to calculate the linear predictor scores (LPS). To remove the systematic differences from two array platforms, we shifted and scaled the expression for each gene from exon array data to the same mean and standard deviation of the gene in the Lymphochip data. Samples were then classified into ABC or GCB subgroup with at least 90% likelihood of being corresponding subgroup. Samples, which were not classified into either of the subgroup were assigned to Type 3.

**Supplementary References**

1. Holte H, Leppa S, Bjorkholm M, Fluge O, Jyrkkio S, Delabie J*, et al.* Dose-densified chemoimmunotherapy followed by systemic central nervous system prophylaxis for younger high-risk diffuse large B-cell/follicular grade 3 lymphoma patients: results of a phase II Nordic Lymphoma Group study. *Ann Oncol* 2013; **24**(5)**:** 1385-1392.

2. IARC. *WHO Classification of Tumours of Haematopoietic and Lymphoid Tissues*, vol. 2. IARC: Lyon, France, 2008, 439pp.

3. Wright G, Tan B, Rosenwald A, Hurt EH, Wiestner A, Staudt LM. A gene expression-based method to diagnose clinically distinct subgroups of diffuse large B cell lymphoma. *Proceedings of the National Academy of Sciences of the United States of America* 2003; **100**(17)**:** 9991-9996.

**Supplementary Figures**

**
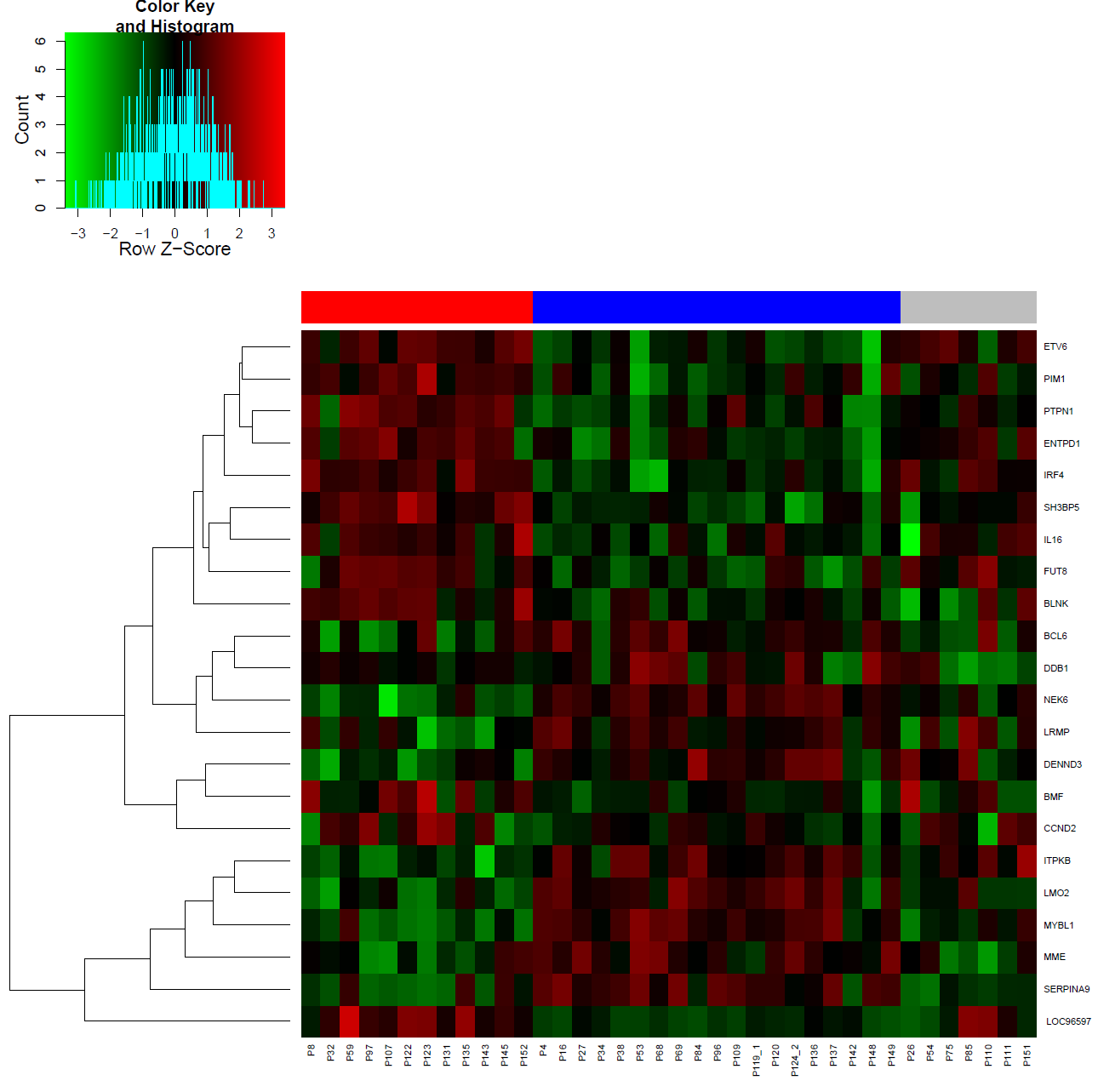
**

**Supplementary Figure S1.** Molecular subtype division according to gene expression profile.

**Supplementary Tables**

**Supplementary Table S1.** Differentially expressed genes (DEGs) between the poor and favorable response groups.

**Supplementary Table S2.** The pathways significantly enriched among the DEGs.

**Supplementary Table S3.** Genes with differentially expressed exons (DEEs) between the poor and favorable response groups.

**Supplementary Table S4.** DEGs between the GCB and the ABC DLBCLs.

**Supplementary Table S5.** DEEs between the GCB and the ABC DLBCLs.

**Supplementary Table S6.** Pathways enriched among the DEGs and DEEs between GCB and ABC subtypes.

**Supplementary Table S7.** Genomic locations of the DEEs.

**Supplementary Table S8.** Alternatively spliced genes common in the discovery and validation cohorts.

**Supplementary Table S9.** Cox multivariate analysis of the DEEs with IPI score for PFS in the validation cohort
