# Supplementary material for: Alternative splicing discriminates molecular subtypes and has prognostic impact in diffuse large B-cell lymphoma
Source: Blood Cancer J. 2017 Aug 25;7(8):e596–. doi: 10.1038/bcj.2017.71 (PMC5596382; doi:10.1038/bcj.2017.71)
Supplement: Supplementary Table 2 [file bcj201771x3.docx]

**Supplementary Table S2. Pathways significantly enriched among the DEGs and DEEs.**

| GENE LEVEL | Pathway Name | Input/  tot# of genes | FDR | Gene symbol |
| --- | --- | --- | --- | --- |
|  | Antigen processing and presentation | 2/89 | <0.001 | HLA-DQB1, CIITA |
|  | Circadian rhythm | 2/13 | <0.001 | ARNTL, PER1 |
|  | Jak-STAT signaling pathway | 7/155 | 0.001 | CCND3, CSF2RB, IL2RB, IL6, IL7, SOCS3, SPRY4 |
|  | Hematopoietic cell lineage | 5/87 | 0.003 | CD55, CR1, IL6, IL7, MME |
|  | Biosynthesis of unsaturated fatty acids | 3/22 | 0.004 | FADS1, FADS2, YOD1 |
|  |  |  |  |  |
| EXON LEVEL | Antigen processing and presentation | 17/89 | <0.001 | HLA-DQA1, CD4, HLA-DQA2, RFXAP, HLA-DMB, HLA-DPB1, HLA-DOA, B2M, TAP2, HLA-F, HLA-B, RFX5, CREB1, PSME2, CANX, PSME1, KIR2DL3 |
|  | Leukocyte transendothelial migration | 22/119 | <0.001 | ICAM1, ACTB, ITGAM, CTNNA1, PIK3R5, ARHGAP5, JAM2, NCF2, PIK3CB, CLDN11, PLCG2, NOX1, CLDN7, THY1, PRKCG, CLDN19, RAPGEF4, CTNNA2, CTNNA3, MAPK13, MAPK12, BCAR1 |
|  | Cell adhesion molecules (CAMs) | 26/134 | <0.001 | HLA-DQA1, ICAM1, CD4, HLA-DQA2, PTPRF, ITGAM, ITGB7, HLA-DMB, ITGB8, HLA-DPB1, HLA-DOA, PTPRM, HLA-F, ALCAM, JAM2, L1CAM, HLA-B, CD34, CLDN11, NRXN2, CLDN7, NRXN1, NLGN1, CNTN1, CNTN2, CLDN19 |
|  | Adherens junction | 19/78 | <0.001 | PARD3, ACTB, PTPRF, SMAD3, TCF7L2, IGF1R, CTNNA1, TJP1, PTPRM, EGFR, TCF7L1, TGFBR2, SNAI1, FYN, CTNNA2, WASF1, CTNNA3, RBB2, SNAI2 |
|  | Phosphatidylinositol signaling system | 21/76 | <0.001 | DGKH, PLCB2, DGKZ, SYNJ2, PLCB1, PIK3R5, PLCB4, SYNJ1, DGKE, INPP5D, INPP5B, CALM1, PLCE1, PIK3CB, DGKD, PLCG2, INPP4B, PRKCG, DGKB, DGKQ, ITPKA |
|  | ECM-receptor interaction | 33/84 | <0.001 | LAMA5, CD44, ITGA7, COL5A1, LAMA4, ITGB7, THBS1, ITGB8, CD36, ITGA3, COL4A2, ITGA2, COL6A2, LAMA3, HSPG2, DAG1, ITGA5, THBS3, COL6A6, CHAD, COL4A4, ITGA1, SV2A, TNC, LAMB3, SV2C, AGRN, COL11A2, TNR, LAMB4, COL4A6, LAMA1, COL5A3 |
|  | Focal adhesion | 59/203 | <0.001 | AKT3, LAMA5, ITGA7, ACTB, FLT1, COL5A1, LAMA4, KDR, CAPN2, IGF1R, ITGB7, PIK3R5, THBS1, ITGB8, TLN1, RAPGEF1, PDPK1, ARHGAP5, EGFR, ITGA3, FYN, COL4A2, TLN2, BIRC2, ITGA2, RAF1, COL6A2, LAMA3, PIK3CB, ITGA5, THBS3, FLNB, COL6A6, CHAD, PDGFB, COL4A4, ITGA1, PARVG, TNC, VEGFA, LAMB3, DIAPH1, DOCK1, CAV3, PRKCG, MAPK10, PAK7, HGF, PPP1CC, MYLK, COL11A2, TNR, LAMB4, COL4A6, LAMA1, ERBB2, COL5A3, BCAR1, FLT4 |
|  | Pathways in cancer | 84/330 | <0.001 | LAMA5, TRAF1, CTBP2, CSF3R, AXIN1, LAMA4, SMAD3, TCF7L2, ARNT2, RALGDS, NOS3, AKT3, PIAS1, IGF1R, PTGS2, CTNNA1, PIK3R5, ITGA3, CASP8, COL4A2, RET, EGFR, ITGA2, VHL, CBLC, TRAF3, RARA, BIRC2, LAMA3, RALB, TCF7L1, APC, TGFBR2, RAF1, NFKB1, PPARD, MAP2K2, PDGFB, TPR, COL4A4, TP53, PML, TFG, PIK3CB, TPM3, WNT10A, RUNX1, VEGFA, BCR, LAMB3, DVL3, PAX8, HHIP, PLCG2, FGF23, NTRK1, NOS2, FGF12, HGF, PTCH2, EGLN3, SMO, DCC, PRKCG, WNT3A, FZD6, HDAC2, KLK3, PIAS4, RXRA, WNT5A, MAPK10, LAMB4, WNT9A, CTNNA2, WNT7B, CTNNA3, NFKB2, WNT4, COL4A6, LAMA1, FGFR2, FGFR3, ERBB2 |
|  | Calcium signaling pathway | 50/182 | <0.001 | PDE1B, PLCB2, CACNA1C, ADCY3, NOS3, ADCY7, ATP2A3, PLCB1, EGFR, PLCB4, HRH2, ADCY4, RYR1, EDNRA, CAMK4, SPHK2, CALM1, PLCE1, PLCG2, CACNA1F, NOS2, CHRM5, GRM5, ERBB3, GRM1, LHCGR, CACNA1A, ATP2B2, ATP2B3, PRKCG, CAMK2G, MYLK, CAMK2A, GRIN1, ERBB4, RYR3, SLC8A2, CACNA1G, P2RX1, BDKRB1, P2RX6, SLC8A3, CACNA1I, RYR2, HTR2C, CHP2, ITPKA, CAMK2B, SLC25A4, ERBB2 |
|  | ABC transporters | 19/44 | <0.001 | ABCA3, ABCA7, ABCA2, ABCA5, TAP2, ABCC9, ABCD3, ABCB1, ABCC3, ABCC10, ABCC5, ABCG5, ABCA10, ABCA4, ABCA8, ABCB5, CFTR, ABCB11, ABCG8 |
|  | MAPK signaling pathway | 64/272 | <0.001 | RASGRF2, IL1B, ARRB2, CACNA1C, TNFRSF1A, MAP3K5, NFATC2, RPS6KA1, MAP2K3, AKT3, RASGRP4, MAP2K6, PTPN5, MAP3K13, GADD45G, EGFR, PLA2G4A, MAP3K4, MAPKAPK2, RASGRP3, TGFBR2, MAP2K5, TAOK1, RAF1, FLNB, NR4A1, NFKB1, MAP3K6, MAP2K2, RPS6KA5, PDGFB, RASA2, DUSP6, TP53, RPS6KA4, NF1, NFATC4, PLA2G2F, FGF23, CACNA1F, NTRK1, CACNA2D4, FGFR4, PLA2G3, CACNG8, FGF12, CACNA1A, CACNG7, PRKCG, DAXX, MAPT, IL1A, CACNA1G, MAPK10, CACNB4, NTF3, CACNA1I, NFKB2, CHP2, MAPK13, FGFR2, FGFR3, RASGRP1, MAPK12 |
|  | Long-term depression | 25/75 | <0.001 | PLCB2, NOS3, IGF1R, GRIA3, PLCB1, PLCB4, PLA2G4A, RYR1, RAF1, MAP2K2, LYN, PPP2R1B, PLA2G2F, GRIA1, NOS2, CRHR1, GRM5, PLA2G3, GRM1, CACNA1A, PRKCG, GRID2, GUCY2D, GUCY2F, GUCY2C |
|  | Tight junction | 36/135 | <0.001 | PARD3, PRKCQ, ACTB, MAGI3, EPB41L2, AKT3, EXOC3, CTNNA1, EXOC4, TJP1, EPB41L3, LLGL2, JAM2, MAGI1, MYH10, AMOTL1, MPDZ, PPP2R1B, SPTAN1, TJAP1, CLDN11, PPP2R2B, LLGL1, MYH13, CLDN7, TJP3, PRKCG, CGN, CLDN19, PRKCE, CSDA, EPB41L1, CTNNA2, CTNNA3, MYH14, PRKCZ |
|  | Amyotrophic lateral sclerosis (ALS) | 19/56 | 0.001 | TNFRSF1A, MAP3K5, MAP2K3, NOS3, MAP2K6, ALS2, NEFM, TNFRSF1B, CASP1, TP53, GRIA1, NOS2, TOMM40, DAXX, GRIN1, RAB5A, CHP2, MAPK13, MAPK12 |
|  | Small cell lung cancer | 25/86 | 0.001 | TRAF1, LAMA5, AKT3, LAMA4, NOS3, PIAS1, PTGS2, NFKB1, TRAF3, BIRC2, PIK3R5, ITGA3, COL4A2, ITGA2, LAMA3, COL4A4, TP53, PIK3CB, LAMB3, NOS2, PIAS4, RXRA, LAMB4, COL4A6, LAMA1 |
|  | VEGF signaling pathway | 22/74 | 0.002 | NFATC1, NFATC1, NFATC2, KDR, NOS3, AKT3, PTGS2, PIK3R5, PLA2G4A, MAPKAPK2, RAF1, MAP2K2, SPHK2, PIK3CB, VEGFA, NFATC4, PLA2G2F, PLCG2, PLA2G3, PRKCG, CHP2,  MAPK13, MAPK12 |
|  | Axon guidance | 33/129 | 0.002 | SEMA4D, PLXNA2, EPHB2, SEMA4C, NFATC1, EPHA3, NFATC2, SRGAP2, SEMA3F, LIMK2, PLXNA1, L1CAM, ARHGEF12, SRGAP3, FYN, LIMK1, NFATC4, SEMA5B, ABLIM2, EPHA5, UNC5C, DCC, PAK7, EPHA6, RND1, SEMA3B, EPHA8, DPYSL5, UNC5A, RGS3, CHP2, NTNG1, NGEF |
|  | Type I diabetes mellitus | 14/44 | 0.002 | HLA-DQA1, HLA-DQA2, IL1B, HLA-DMB, HLA-DPB1, HLA-DOA, HLA-F, ICA1, HLA-B, PTPRN, GAD1, IL12A, IL1A, GAD2 |
|  | Regulation of actin cytoskeleton | 47/217 | 0.003 | ARHGEF1, ITGA7, ACTB, IQGAP2, CYFIP1, ITGAM, TIAM1, ITGB7, ITGB8, PIK3R5, ITGA3, EGFR, ITGA2, APC, ITGA5, SSH1, RAF1, DIAPH1, LIMK2, MAP2K2, PDGFB, MYH10, ARHGEF12, ITGA1, DOCK1, PIK3CB, LIMK1, ARPC2, SSH3, ARPC5L, FGF23, CHRM5, FGD1, FGFR4, SCIN, FGF12, PPP1CC, PAK7, RDX, MYLK, BDKRB1, IQGAP3, WASF1, FGFR2, FGFR3, MYH14, BCAR1 |
|  | Thyroid cancer | 11/29 | 0.003 | TCF7L2, RET, TCF7L1, MAP2K2, TPR, TP53, TFG, TPM3, PAX8, NTRK1, RXRA |
